# A 10-Year Single Center Analysis: Is There a Need to Reconsider Our Approach to the Management of Neonatal Testicular Torsion?

**DOI:** 10.7759/cureus.74350

**Published:** 2024-11-24

**Authors:** Luke J Turner, Alison Campbell

**Affiliations:** 1 Pediatric Surgery, Royal Hospital for Children, Glasgow, GBR

**Keywords:** asynchronous testicular torsion, bilateral testicular torsion, emergency bilateral exploration, neonatal testicular torsion, perinatal testicular torsion

## Abstract

The management of neonatal testicular torsion lacks consensus. Arguments in favor of emergency bilateral exploration and fixation include a salvage rate and the incidence of bilateral torsion. We performed a retrospective single-center analysis of all cases of neonatal torsion in our unit between 2012 and 2022 to assess whether our data supports this approach and to publish further data on a disease on which there remains a paucity. During this period, there were 28 neonates with suspected torsion - 24 had torsion.

The majority, 25 (89%), of patients were explored, with 12 (48%) of these as an emergency (<6 hrs of surgical review). Nineteen (95%) of those who had the symptomatic side explored also had a contralateral exploration. Three (15%) of operated testes were salvaged - one atrophied. None of the contralateral fixed testicles atrophied and there were no anesthetic complications. One patient (4%) had bilateral torsion - one testicle was asymptomatic and chronically infarcted, and the symptomatic testis was not salvageable, leading to anorchia.

We conclude that neonatal torsion has a salvage rate, and bilateral exploration and fixation are low risk. Moreover, we present a rare case of bilateral torsion and an asymptomatic contralateral torsion - pediatric surgeons must be made aware of such cases as delaying treatment of the symptomatic testicle could result in the devastating outcome of anorchia.

## Introduction

Neonatal testicular torsion (NTT) management lacks consensus. The primary controversies are whether to explore the suspected torted side or not, whether to explore the contralateral side, and whether to do either as an emergency. In 2011, among pediatric surgeons in the UK, 25% did not explore, 28% did not explore the contralateral side and even fewer undertook emergency exploration [1]. This may have been due to a paucity of robust literature concerning the topic. Our center was an early advocate for emergency exploration because of the risk of bilateral torsion and the potential salvage rate [2]. However, the paucity of data on the incidence of bilateral torsion led some authors to prefer to delay contralateral exploration and fixation [3], whereas others did so as an emergency [4].

Over the past decade, more substantial evidence to guide practice has emerged. Salvage rates as high as 21.7% [5] and the incidence of bilateral torsion as high as 14.9% [6] have been reported. Furthermore, a large international study of long-term developmental outcomes following anesthesia in neonates and infants has suggested no significant difference in developmental outcomes at starting school [7]. This may provide some reassurance regarding anesthesia in neonates.

The purpose of this study was to perform a 10-year analysis of all of the cases of neonatal torsion in our center, to add to what is a growing but still very limited international data set, and to see if our data supports the policy of emergency bilateral exploration.

## Materials and methods

All cases in our unit from 2012 to 2022 were reviewed regarding the following.

Time of symptom onset

The patient’s medical notes were reviewed and the first documented description of any testicular signs or symptoms was taken at the time of onset.

Time from review to operation

The patient’s medical notes were reviewed and the timing between the surgical review and their operation was assessed. This was chosen to be measured, as opposed to time from symptom onset to operation, for several reasons. Firstly, the timing of symptom onset, as assessed retrospectively, is unreliable, given some neonates may only have had their testicles checked for the first time during their newborn physical screening examination (up to 72 hours after birth) and therefore may have been symptomatic prior to it being formally documented. In addition, only one case in our study was taken to the theater within six hours of symptom onset, so any meaningful statistical analysis of this parameter would have been very limited.

Contralateral exploration

The patient’s operation notes were reviewed and assessed to see whether they underwent unilateral or contralateral exploration.

Incidence of contralateral torsion

The patient’s medical notes and operation notes were reviewed to see if there was any documented evidence of a contralateral torsion, as diagnosed clinically, radiologically, or intra-operatively.

Anesthetic complications

The patient’s medical notes, namely the anesthetic chart, operation note, post-operative review, and outpatient clinical follow-up notes, were assessed for any immediate or long-term anesthetic complications. Salvage rate and atrophy rate - these two parameters were assessed by reviewing the operation notes to see which torted testicles underwent orchidopexy. The outpatient follow-up notes for these patients were then reviewed and if the surgeon deemed the testicular volume to be ≥50% of the contralateral, healthy testicle, then the testicle was deemed to have been salvaged. If the testicle was deemed to be ≤50% of the volume of the contralateral, healthy testicle, then it was deemed to have atrophied. In the existing literature, there does not appear to be an accepted testicular volume that would quantify a successful salvage versus an atrophy specifically for neonatal torsion; none of the three large literature reviews on the topic offer such a value [5,6,8]. However, the value seems to vary between 25% and 50% of the contralateral testicle [9,10]. These metrics were used for all of the cases of unilateral torsion - in one case of bilateral torsion, the surgeon was not granted such a comparison and so had to decide on whether the operated testicle was of a normal physiological volume based on their clinical expertise.

Additionally, patient demographics were extracted from their medical notes, including gestational age, birth weight, and the presence of co-morbidities. The cases were found using two of our hospital databases - Badgernet, a neonatal database for all neonates admitted to the hospital, and Opnote Archive, a database for all patients who have had surgery. For Badgernet, searches were performed using the word “torsion” under the “diagnosis” search and, independently, “other search terms” - this yielded the notes of any neonate whose medical notes included the word “torsion” between our search dates. Using Opnote Archive, all operations containing the words “testicle”, “testicular”, “scrotum”, “scrotal” or “torsion” were reviewed for cases of neonatal scrotal explorations. Reassuringly, no cases were found in the latter search that had not already been found in the former. Patients were excluded if they were managed non-operatively and did not have definitive ultrasound evidence of torsion or if they were over 30 days old at the time of presentation. Statistical analysis was performed using Fisher’s exact test.

In order to minimize bias and improve data reliability, both authors gathered the data independently, and where there were any disputes, a third-party opinion was sought. The data were then presented locally to ensure that the wider surgical team, including the operating surgeons involved in the presented cases, were satisfied with the data integrity.

## Results

Pathology

Between 2012 and 2022, there were 28 cases of suspected NTT at our center. Of these, 25 were taken to the theater in which 18 had torsion, two were suspected to have recently been torted but untwisted (dusky without other cause), four were not torted (three hematomas, one epididymo-orchitis) and one only had the contralateral side explored. However, the ipsilateral side was atrophied at follow-up and so was likely to have been torted. Assuming this latter case and the two dusky testicles were torsions, there was a total of 21 (84%) torsions of the 25 taken to the theater. Nine were extravaginal, one was intravaginal and 11 were not specified. Three not taken to the theater were proven to be avascular on ultrasound - assuming this was secondary to torsion, 24 (85.7%) of the 28 cases of suspected torsion proved to be torsion. A summary of these findings can be seen in Table 1.

**Table 1:** Pathology findings

Pathology findings	Number of patients
Intra-operative finding of torsion	18 (64.3%)
Intra-operative suspicion of torsion	2 (7.1%)
Ultrasound-proven torsion, not taken to theater	3 (10.7%)
Ultrasound-proven torsion, only contralateral side explored	1 (3.6%)
Haematoma/bruising	3 (10.7%)
Epididymo-orchitis	1 (3.6%)

Epidemiology

Out of 24, 22 (91.7%) of the neonates with torsion were born at term, and two (8.3%) were premature. Of 24, one (4.17%) was underweight (<2,500g), 18 were of a normal birth weight, and birth weights of five could not be found. Of 24, three (12.5%) had co-morbidities - these were jejunal atresia, empyema, and respiratory distress.

Timing of symptoms

Seventeen (70.8%) had signs at birth (antenatal). Of the seven (29.2%) who developed signs postnatally, three (42.9%) developed signs in the first 24hrs and four (57.1%) developed symptoms later on.

Laterality

Of the 24 cases of torsion, seven (29.17%) were left-sided, 16 (66.67%) were right-sided, and one (4.17%) was bilateral. The bilateral case was asynchronous, given that only one side was symptomatic, and the contralateral side was chronically infarcted on ultrasound at the time of presentation. The patient was taken to the theater for a unilateral exploration of the symptomatic side, but unfortunately, the testicle was not viable and an orchidectomy was performed. At follow-up, the patient’s contralateral, chronically infarcted testicle remained as a nubbin and the patient is now anorchic.

Surgical approach

25/28 (89%) were explored surgically. Of these, 12 (48%) were taken to the theater as an emergency (within six hours of being reviewed by the surgical team), 11 (44%) were operated on semi-urgently (on an emergency theater list during the same admission, but after six hours of being reviewed by the surgical team) and two (8%) were operated on electively (at three- and four-week post-review). Four (57%) postnatal torsions were taken to the theater as an emergency, compared with eight (47%) antenatal torsions.

Of the three (11%) that were not taken to the theater, one neonate was too sick to safely take them to the theater due to co-morbidities, and when stabilized, an ultrasound showed a return of blood flow to the testicle. One was also too sick for theater at the time and upon stabilization, their surgeon deemed them to have moved out of the high-risk stage for a contralateral torsion and so they were not explored. One of the three was healthy at birth but their surgeon opted not to take them for an exploration due to personal preference.

**Figure 1:**
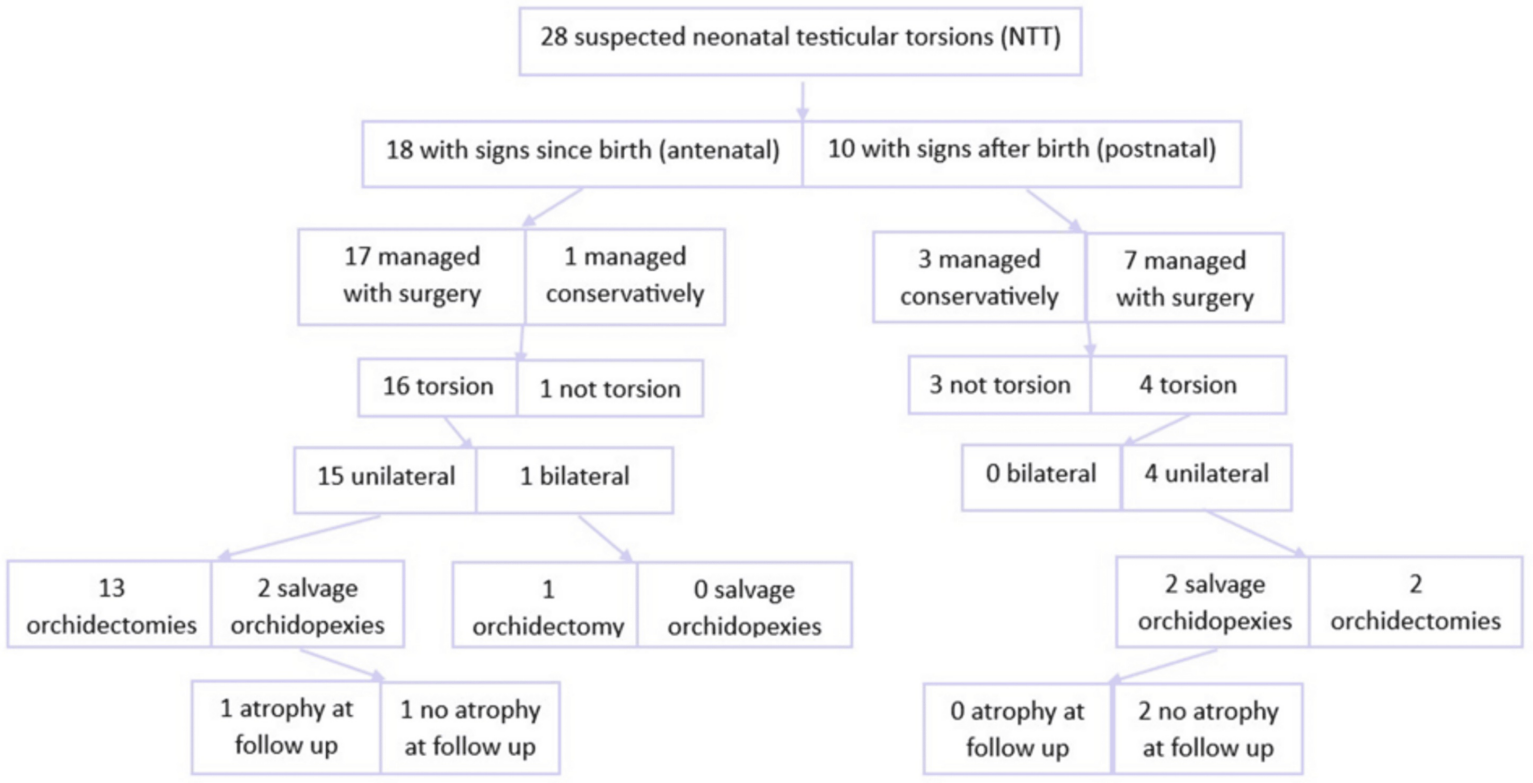
Surgical approach and outcomes

Of the 25 patients taken to the theater, 21 patients were found to have, or presumed to have, torsion - 20 (95%) had an exploration of the symptomatic side, and one (5%) had a contralateral exploration and fixation only. Of the 20 that had an exploration of the symptomatic side, 16 (80%) had an orchidectomy and four (20%) had an orchidopexy. Furthermore, of the 20 that had an exploration of the symptomatic side, 19 (95%) had a contralateral exploration and fixation. The one that did not have a bilateral asynchronous torsion with a chronically infarcted contralateral testicle was shown on ultrasound. A summary of these findings can be seen in Figure 1.

Change in practice over time

**Figure 2:**
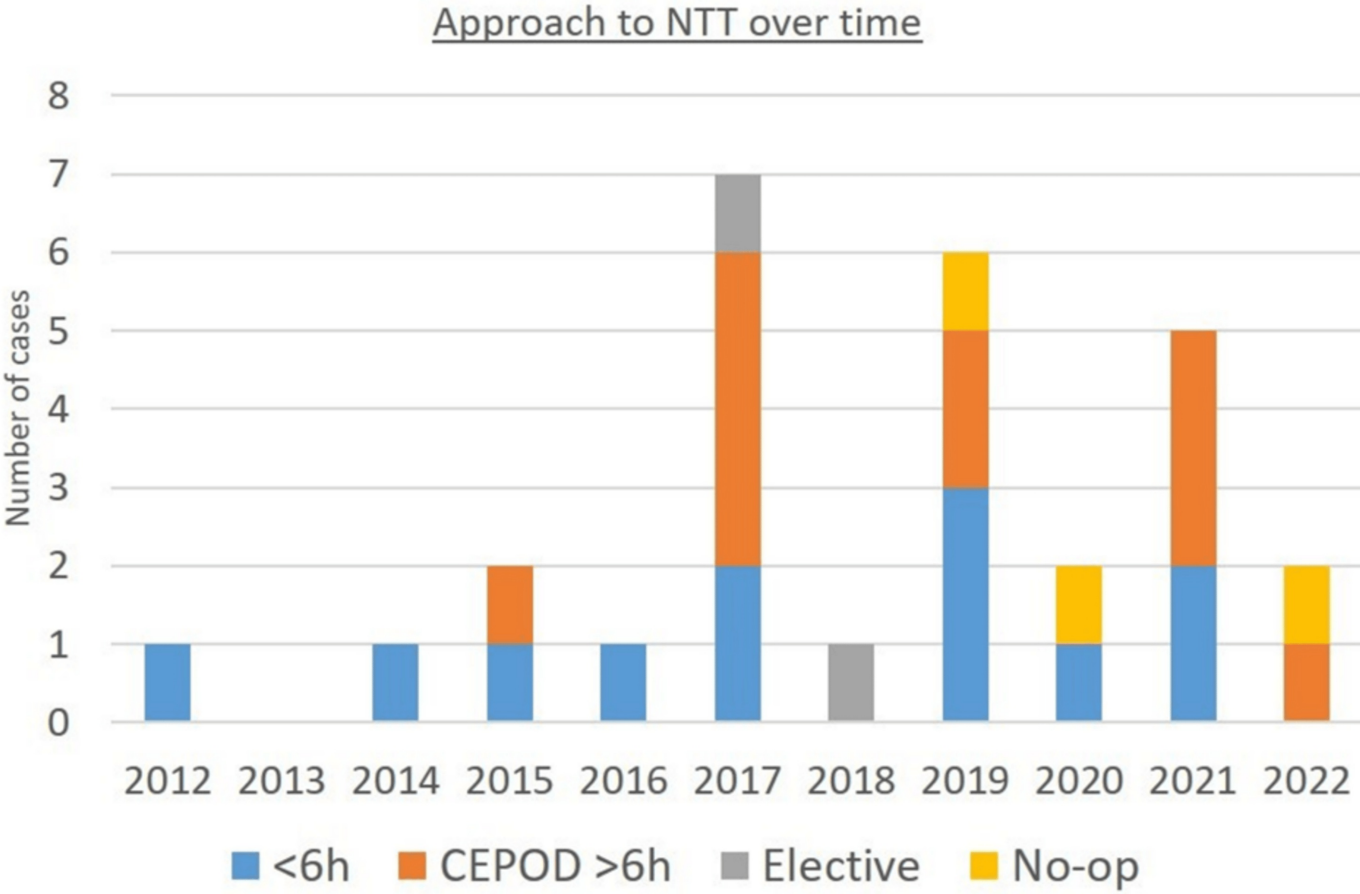
Change in practice over time *NTT – neonatal testicular torsion, *6h – 6 hours, *CEPOD – confidential enquiry into perioperative deaths (CEPOD theater means emergency theater in the UK)

The surgical approach to neonatal torsion in our center over the decade in the study can be seen in Figure 2. It was difficult to reliably elucidate if there had been any change in practice over time, given that the caseload in some years was minimal (and even non-existent in one year). Although it could be argued that a larger proportion of cases were taken as an emergency in the early years, it may just be that we see the true approach in the years with more cases, i.e., the later years. Except for 2022, looking at only those years in which there were at least two cases, the fraction that was taken as an emergency does not appear to have changed over the course of the decade.

USS and tumors

None of the 28 cases were clinically concerning for a testicular tumor - as such, surgery was not delayed in order to carry out imaging to rule out a tumor for any of the cases. Of the three not taken to the theater, all had an ultrasound which did not show a tumor. Of the 25 taken to the theater, none were found to have a tumor.

Salvage rate

Of the 21 torsions that were taken to the theater, 20 had the symptomatic side explored. Of these, 19 were taken to theater urgently or semi-urgently (see above for definitions). Of these, three (15.8%) patients had their torted testicles successfully salvaged - all were unilateral torsions.

Two (22.2%) out of nine taken as an emergency were salvaged compared to one out of 10 (10%) taken semi-urgently (p=0.582). Three out of 15 (20%) were taken to the theater in <48 hours since symptom onsets were salvaged compared to zero (0%) taken >48 hours (p=1.0). Further analysis of the salvage rate based on emergency v non-emergency and antenatal v post-natal torsion can be seen in Table 2.

**Table 2:** Salvage rate *Although 21 cases of torsion were taken to theater, only 19 were taken non-electively.

	All cases	Antenatal	Postnatal
Overall salvage rate	15.8% (3/19*)	6.7% (1/15)	50% (2/4)
Salvage rate if taken to theater <6h post-review	22.2% (2/9)	14.3% (1/7)	50% (1/2)
Salvage rate if taken to theater >6h post-review	10% (1/10)	0% (0/8)	50% (1/2)
Salvage rate if taken to theater <48h since symptom onset	20% (3/15)	10% (1/10)	50% (2/4)
Salvage rate if taken to theater >48h since symptom onset	0% (0/4)	0% (0/4)	N/A

It should be noted that the one case of antenatal torsion that was salvaged was one of the two cases in which the testicle was found to be grey/dusky intra-operatively but without a visualized twist. An ultrasound prior to the exploration revealed the testicle to be echogenic with a hypoechoic rim. Given the focality of the ischemia (the patient had no other hematological disorder or other focus of ischemia), we have assumed that this represented a recently torted but untwisted testicle.

It is also important to note that one of the two postnatal cases that were salvaged was found to have pus in the scrotum, which made anatomical observations difficult. However, the operating surgeon did report observing a 180-degree extra-vaginal torsion.

Complications

Of the 25 patients taken to the theater, an anesthetic record was available for 23 of them, all of which had no anesthetic complications. The surgery was non-therapeutic for four (16.7%) of them (those without torsion). Follow-up was found for 18 of the 25. No complications were found at follow-up, but one out of the four (25%) patients that had an orchidopexy of a torted testicle experienced atrophy at follow-up.

## Discussion

Our study spanned 10 years of practice and included 24 patients with torsion. Over this period, our practice was that of surgical exploration, primarily that of bilateral exploration and contralateral fixation. Just under half of operations were performed within six hours of review. This practice did not change over the course of the period studied and may have been influenced by our own case series recommending urgent exploration published in 2007 [2].

Bilateral torsion represents between 7.1% and 14.9% of cases in the literature reviewed. It is unclear whether synchronous or asynchronous bilateral torsion is more common. The median time between torsions in asynchronous torsion is one day [6]. Bilateral torsion can present with unilateral signs.

We found the rate of bilateral torsion to be one out of 24 (4.17%) - slightly lower than the wider literature but almost certainly an underestimate given that of the 24 cases of torsion, 18 (75%) of these were cases of unilateral torsion that had a contralateral fixation performed emergently, preventing the possibility of an asynchronous torsion. This is not the standard practice elsewhere [11]. In our series, there was one case of a “silent” asynchronous torsion that led to anorchia.

In a more recent meta-analysis [8] and literature review [6], overall salvage rates varied between 7.3% and 9%. Our overall initial salvage rate was three of 20 (15%). This is greater than that reported in the studied literature and may be due to our small sample size or due to the fact that almost half of the cases were taken as an emergency, a greater fraction than elsewhere reported. Indeed, Nandi et al. reported that the salvage rate was greater for those taken as an emergency (22.2%) [5]. This salvage rate may be an underestimation of the potential salvage rate of NTT if taken to the theater within six hours of symptom onset, as of all of our cases taken to the theater, only one was definitely taken within six hours of symptom onset although 48% were taken within 6hrs of surgical review. It is challenging to reliably and accurately ascribe a time of symptom onset from a retrospective analysis of the notes alone, so this parameter was not formally included in the analysis.

We report a salvage rate of one of 15 (6.7%) for antenatal torsion, up to one of seven (14.3%) if taken as an emergency. This is significant as the historical consensus was that only post-natal torsion had a salvage rate [3,12,13].

We had three cases of possible torsion-detorsion. One was found to display signs of torsion on a pre-operative ultrasound and to be ischemic intra-operatively, no actual twist of the cord/tunica vaginalis was seen, this was pexed and did not atrophy at follow-up. One case of an ischemic testicle intraoperatively without a visible twist was not able to be salvaged. We also had a case of torsion in a neonate, proven on ultrasound, not taken to the theater acutely due to being too unwell. On stabilization 17 days later, a repeat ultrasound was performed and the testicle’s vascularity returned. This case appears to be a first in the published literature that we can see, showing spontaneous recovery of an ischemic neonatal testicle on serial ultrasounds. In light of these three cases, we hypothesize that NTT can sometimes “un-twist” after the initial tort. The majority of neonatal torsions are extravaginal. The fascial layers have not attached to each other or the dartos [14] and this absence of anchoring may make it easier to untwist. Exploring NTT as an emergency, therefore, even if present antenatally, might be of benefit. In two of the above three cases, the testicle may not have been irreversibly ischemic due to twisting and untwisting and could benefit from urgent fixation to prevent further events.

There were no anesthetic complications recorded for any of the patients in our study. There were no cases of atrophy of the contralateral pexed testis on follow-up. We did not have any patients operated on emergently for suspected torsion diagnosed with a testicular tumor at the time of exploration. Therefore, emergent bilateral exploration and fixation in neonatal torsion do not seem to pose a significant risk to the otherwise well-term neonate.

Limitations to the study

There were multiple limitations to this study. Firstly, it was a single-center study and as such the surgical technique employed, in addition to the patient demographic, may not be reflective of the wider population of surgeons and patients. In addition, it was a retrospective study. This applies the constraint of rendering it difficult to accurately ascertain data, such as the timing of onset of symptoms, the time between onset of symptoms and review/surgery, the rationale for exploring/not exploring, and the patient’s clinical picture. As per the materials and methods section, steps were taken to minimize bias and improve data reliability.

Finally, the small sample size of the study made it underpowered and therefore difficult to make conclusions with statistical significance. Unfortunately, this is a common theme amongst neonatal torsion studies, due to the rarity of the condition. Indeed, in the largest pooled data analyses on the topic - the literature reviews by O’Kelly et al. [6] and Nandi et al. [5], in addition to the meta-analysis by Monteilh et al. [8], the average number of cases per a study in the studies included in the analyses were nine, 15 and 22, respectively - all fewer than in this study. We anticipated that the study would be underpowered, but as the analysis included all available data since the advent of the databases used in our center, this was not something that we would be able to change and as such we did not perform a priori power analysis. However, moving forward, we will aim to pool our data with other centers in order to improve statistical power.

## Conclusions

Emergency exploration offers potential salvage in postnatal torsion and a smaller but important chance of salvage in antenatal torsion. Emergency exploration may be of particular importance given the risk of a pre-existing contralateral, asynchronous, “silent” torsion, as the symptomatic side would then represent the only viable testicle and all salvage attempts should therefore be taken urgently. A future potential research direction would be to assess if ultrasound analysis at the time of presentation could help to identify more of such cases, and as such encourage more surgeons to explore the remaining, symptomatic side as an emergency. Furthermore, emergency contralateral exploration and fixation mitigates the small but catastrophic risk of subsequent contralateral, asynchronous torsion that would otherwise likely lead to anorchia. Moving forward, we will look to pool our data with other centers, in order to increase the statistical power of our analysis and further reiterate the gravity of the above conclusions.
